# Beneficial Effects of Dietary Nitrate on Endothelial Function and Blood Pressure Levels

**DOI:** 10.1155/2016/6791519

**Published:** 2016-03-21

**Authors:** Jenifer d'El-Rei, Ana Rosa Cunha, Michelle Trindade, Mario Fritsch Neves

**Affiliations:** Department of Clinical Medicine, State University of Rio de Janeiro, 20551-030 Rio de Janeiro, RJ, Brazil

## Abstract

Poor eating habits may represent cardiovascular risk factors since high intake of fat and saturated fatty acids contributes to dyslipidemia, obesity, diabetes mellitus, and hypertension. Thus, nutritional interventions are recognized as important strategies for primary prevention of hypertension and as adjuvants to pharmacological therapies to reduce cardiovascular risk. The DASH (Dietary Approach to Stop Hypertension) plan is one of the most effective strategies for the prevention and nonpharmacological management of hypertension. The beneficial effects of DASH diet on blood pressure might be related to the high inorganic nitrate content of some food products included in this meal plan. The beetroot and other food plants considered as nitrate sources account for approximately 60–80% of the daily nitrate exposure in the western population. The increased levels of nitrite by nitrate intake seem to have beneficial effects in many of the physiological and clinical settings. Several clinical trials are being conducted to determine the broad therapeutic potential of increasing the bioavailability of nitrite in human health and disease, including studies related to vascular aging. In conclusion, the dietary inorganic nitrate seems to represent a promising complementary therapy to support hypertension treatment with benefits for cardiovascular health.

## 1. Introduction

Hypertension is a multifactorial condition characterized by high and sustained levels of blood pressure (BP). It is the most common condition in primary care and often associated with functional and/or structural changes in target organs and metabolic disorders, increasing the risk of fatal and nonfatal cardiovascular events [1, 2]. Among the risk factors for mortality from cardiovascular disease (CVD), hypertension explains 40% and 25% of deaths from stroke and coronary artery disease (CAD), respectively [3].

BP is a biological and dynamic variable, dependent on many factors. Endothelial cells of the vascular system are responsible for many biochemical reactions that maintain vascular homeostasis and consequently the BP levels [4]. Endothelium modulates vascular tone, not only by producing vasodilator substances but also by releasing vasoconstrictive substances through prostanoid of endothelin generation, as well as through conversion of angiotensin I (AI) in angiotensin II (AII) on the endothelial surface. These vasoconstrictor agents not only act mainly locally but also present some systemic effects, playing an important role in regulating the vascular function and remodeling the arterial wall. In healthy individuals, there is a balance among these substances, tending to vasodilatation when endothelial function is normal [5].

## 2. Nitric Oxide (NO), Endothelial Dysfunction, and Metabolic Syndrome

Changes in endothelial function precede morphological changes of blood vessels and contribute to the development of clinical complications of cardiovascular diseases. Thus, the beginning and the clinical course of adverse cardiovascular events depend directly on changes in vascular biology. Lately, it became clear that the endothelium is responsible for promoting vascular homeostasis [5].

The limited NO bioavailability is the main mechanism involved in endothelial dysfunction, which is crucial for the development of CVD [6]. In fact, endothelial dysfunction in peripheral and coronary vessels is an independent predictor of cardiovascular events and represents an early stage of CAD [7]. As endothelial dysfunction is reversible, early detection and intervention could have critical therapeutic and prognostic implications for patients with risk for, or even with established, CVD [7, 8]. Therefore, an improvement in the NO bioavailability can have a major effect on endothelial function and, consequently, on CVD prevention and treatment [9, 10].

Metabolic syndrome can be considered a clinical and biochemical expression of insulin resistance, representing a clustering of central obesity, hypertension, hyperglycemia, and dyslipidemia [19, 20]. Recently, experimental models and clinical studies demonstrated that reductions in the NO bioavailability play a central role in the pathophysiology of metabolic dysfunction. Endothelial nitric oxide synthase- (eNOS-) deficient mice were able to develop high BP and metabolic dysfunction, and both might be the result of insulin resistance [21, 22]. In the same experimental model, several features of metabolic syndrome could be reversed by dietary supplementation with sodium nitrate. The amount of dietary nitrate used for this effect was comparable to those derived from eNOS under normal conditions, which corresponds to a rich intake of vegetables for humans. Besides, dietary nitrate was able to increase tissue and plasma levels of bioactive nitrogen oxides. Lastly, chronic nitrate supplementation prevented the prediabetic phenotype in these animals by reducing visceral fat accumulation and circulating levels of triglycerides [23]. In humans, eNOS polymorphisms have been associated with insulin resistance, type 2 diabetes mellitus, and metabolic syndrome [21]. Furthermore, recent evidences have shown that obese subjects present a reduced ability to produce NO [10, 24]. Therefore, the dietary nitrate has been widely studied in clinical trials as an alternative form of the classical pathway of L-arginine to NO production.

## 3. Sources and Beneficial Effects of Dietary Nitrate

Poor eating habits may be considered as risk factors for CVD. In fact, high intake of foods rich in cholesterol, lipids, and saturated fatty acids and low consumption of fiber sources are related to dyslipidemia, obesity, diabetes mellitus, and hypertension [25]. Thus, nutritional interventions associated with changes in lifestyle are recognized as important strategies for primary prevention of hypertension and are auxiliary to pharmacological therapies to reduce cardiovascular risk [26].

Epidemiological evidences suggest that vegetable consumption reduces BP and risk of CVD [27–29]. DASH (Dietary Approach to Stop Hypertension) eating plan is one of the major effective strategies for prevention and nonpharmacological management of hypertension [30]. This eating proposal highlights the importance of increasing fruit and vegetables intake [31], and recent research suggests that the beneficial effects of DASH plan on BP are related to high inorganic nitrate content of food included in this eating plan (e.g., green leaves and root vegetables) [32, 33].

Beetroots, lettuce, chard, arugula, and spinach are the vegetables containing the highest amount of nitrate, >250 mg nitrate/100 g [32].  Table 1 shows the vegetables classification according to the nitrate content. Beetroot is a vegetable, particularly rich in inorganic nitrate, which contains an average of 2056 mg of nitrate in a traditional cultivation. There are some studies using beetroot to test the effects of inorganic nitrate intake on BP [18].

Nitrate (NO_3_^−^) and nitrite (NO_2_^−^), present in beetroot and in other food sources, were recently related to cardiovascular benefits. However, they were previously considered as toxic compounds due to the development of malignancies such as gastric cancer. Therefore, strict rules regarding these inorganic anions are regulated in food and in drinking water [34].

Beetroot and other vegetables sources of nitrate contain approximately 60–80% of the daily nitrate intake in the western population [32]. Nitrate content in vegetables may be influenced by factors related to the plant itself, such as variety, species, and maturity, and to the environment, such as temperature, light intensity, lack of some nutrients, and fertilizer use [11].

International organizations indicate that the consumption of dietary nitrate is about 31 to 185 mg/day in Europe and 40 to 100 mg/day in USA [35], and the oral bioavailability of dietary nitrate is 100% [36]. In 1962, World Health Organization (WHO) set an upper limit of nitrate consumption in food. An acceptable daily intake is 3.7 mg NO_3_^−^/body weight (kg), which is the same value adopted by the European Authority for Food Safety. This amount is equivalent to 300 mg/day for an adult weighing 80 kg [37]. However, there is no evidence that nitrate intake is carcinogenic in humans. Instead, epidemiological evidence indicates that consumption of vegetables reduces risk of cancer [38].

**Figure 1:**
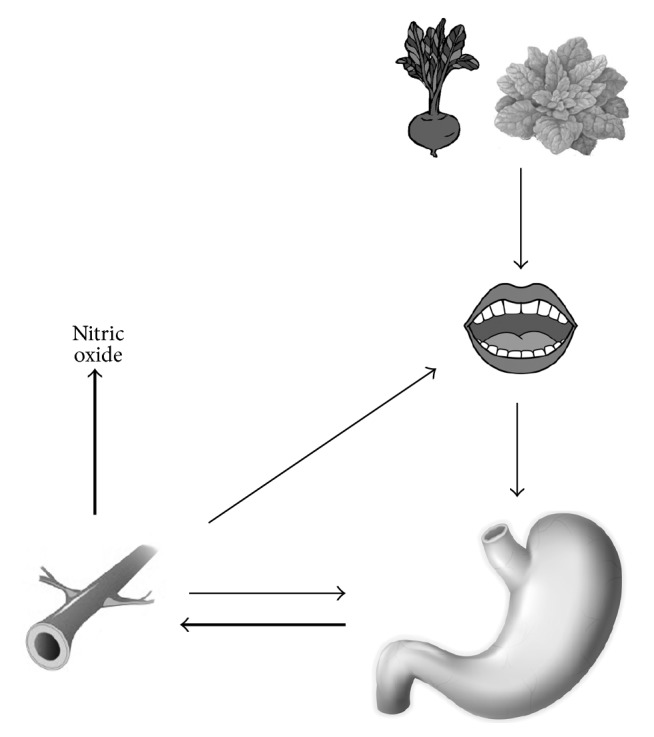
Nitrate (thin arrows) intake from the diet is swallowed and completely absorbed in the upper gastrointestinal tract. About 25% of this amount is concentrated in the salivary glands and still inside the mouth is reduced to nitrite by anaerobic bacteria present on the tongue and is swallowed again. In the stomach, nitrite acid undergoes reduction and is converted to NO (thick arrows) that has vasodilatory action on blood vessels.

After intake, dietary nitrate quickly increases in plasma, in about 30 minutes, reaching its peak in 90 minutes. In contrast, nitrite levels are considerably slower in circulation, reaching their peak in 2.5 to 3 hours. Most of inorganic nitrate, about 75% of absorbed nitrate, is excreted in urine and 25% of plasma nitrate is excreted in saliva [18, 39]. The exact mechanism of salivary concentration is unknown. As a result, there is supply of substrate for nitrate reductase expressed by bacteria that colonize the dorsal surface of the tongue, resulting in reduction of nitrate to nitrite. After nitrite is then swallowed, the stomach and the acid environment reduce it to NO. The remaining nitrite is reabsorbed again by the vascular flow [40]. NO and nitrite continue through the systemic circulation, and the remaining nitrite is reduced to NO in high resistance vessels, promoting vasodilatation and consequently lowering BP [18] (Figure 1). Both NO_3_^−^ and NO_2_^−^ from diet and via L-arginine participate in the NO synthesis [41]. Lately, there is a growing body of interest on the role of these two anions in biological function. The improvement in vascular dysfunction and in BP levels after dietary nitrate seems to be mediated by effects on oxidative stress and inflammation [42, 43].

## 4. Effects of Dietary Nitrate on Blood Pressure

After an acute intake of beetroot juice (500 mL), it is possible to observe reduction of 10 mmHg in systolic BP (after 2.5 h) and reduction of 8 mmHg in diastolic BP (after 3 h) in healthy individuals. The decrease in BP was sustained after 24 h of juice intake. The highest reduction in BP is correlated to the peak in plasma nitrite [18].

Liu et al. evaluated the effects of a meal rich in nitrate (based on spinach consumption) on BP and arterial stiffness in healthy individuals. Two hours after a nitrate-rich meal consumption (220 mg nitrate), there was a larger artery elasticity index, lower pulse pressure, and lower systolic BP compared to the values after a standard meal, low in nitrate [44].

Recent experimental studies [23, 45, 46] and clinical trials have shown that nitrate dietary intakes from beetroot juice [18, 47], beet-enriched bread [48], or inorganic nitrate supplements [49] have a protective effect against CVD because of reducing BP, platelet aggregation inhibition, and prevention of endothelial dysfunction.

Recently, Bondonno et al. have shown that chronic ingestion of beetroot juice (one week, 420 mg nitrate/day) did not improve the BP control in treated hypertensive patients [13]. In another study with overweight elderly subjects, Jajja et al. showed reduction of 7 mmHg in systolic BP, after three weeks of beetroot juice intake (350 mg nitrate/day). However, when BP was evaluated for 24 hours by ambulatory blood pressure monitoring (ABPM), no significant changes were shown in BP levels [14].  Table 2 shows some clinical trials that evaluated the effects of nitrate intake on BP and vascular function. In fact, there is no consensus about the effects of dietary inorganic nitrates on BP and endothelial function, and their effects on cardiovascular health, despite studies with positive results.

Since the initial investigations in healthy volunteers, studies using inorganic nitrate and formulations with nitrate salt showed promising results reducing BP, with nitrate doses ranging from 155 to 1484 mg/day, between 1 and 15 days, with reductions of 4 mmHg in systolic BP and of 1 mmHg in diastolic BP [33, 50]. In hypertensive patients, systolic BP remained significantly reduced in approximately 8 mmHg over 24 hours after the intervention, which is similar to the reduction provided by drug therapy (9 mmHg). This is important because the ingestion of dietary nitrate in a single dose per day may be sufficient to achieve benefits in lowering BP [50, 51].

## 5. Conclusion

Increasing nitrite levels by nitrate intake appears to have beneficial effects in many physiologic and clinical settings. Several clinical trials are being conducted to determine the great therapeutic potential of increasing the bioavailability of nitrite in human health and disease, including studies related to vascular aging. Nevertheless, there are many limitations in nitrate studies, such as the type of population enrolled in each trial and the dependent effect of the baseline BP. Therefore, the effects are unlikely to be the same among healthy and hypertensive individuals. In addition, when evaluating treated hypertensive patients, use of medications, such as calcium channel antagonists, may affect endothelial function and hence can interfere in some vascular parameters. Sample size is also a limiting factor considering the fact that it is small in most of nitrate studies. Indeed, large clinical trials are necessary to confirm the potential beneficial effects of inorganic nitrate in patients with CVD. Even with these considerations, dietary nitrate seems to represent an inexpensive and a promising complementary therapy to support hypertension treatment with benefits for cardiovascular health.

## Figures and Tables

**Table 1:** Vegetables grouping according to the nitrate concentration [11].

Nitrate content (mg/100 g of fresh food)	Vegetables
Very low, <20 mg	Asparagus, garlic, onion, green bean, pepper, potato, sweet potato, tomato, and watermelon
Low, 20–<50 mg	Broccoli, carrot, cauliflower, and chicory
Regular, 50–<100 mg	Cabbage, turnip, and dill
High, 100–<250 mg	Endive, sweet leaf, parsley, and leek
Very high, >250 mg	Celery, chard, lettuce, beetroot, spinach, arugula, and watercress

**Table 2:** Clinical trials evaluating the effects of dietary inorganic nitrate on cardiovascular health.

Population	Study design and duration	Nitrate dose	Control	Vascular parameter	Result	Reference
Hypertensive *n* = 68 57 years 144/88 mmHg	Chronic 4 weeks	450 mg (250 mL beetroot juice)	Beetroot juice poor in nitrate	PWV FMD SBP DBP	↓ PWV, ↑ FMD ↓ SBP, ↓ DBP ↓ SBP_24 h_ ↓ DBP_24 h_	Kapil et al., 2015 [12]

Hypertensive *n* = 27 63 years 133/77 mmHg	Chronic 1 week	420 mg (140 mL beetroot juice)	Beetroot juice poor in nitrate	SBP DBP	No effect	Bondonno et al., 2015 [13]

Overweight elderly *n* = 24 63 years	Chronic 3 weeks	±350 mg (70 mL beetroot juice)	Blackcurrant juice	SBP DBP	↓ SBP No effect on DBP	Jajja et al., 2014 [14]

T2DM *n* = 27 67 years 143/81 mmHg	Chronic 2 weeks	500 mg (250 mL beetroot juice)	Beetroot juice poor in nitrate	FMD	No effect	Gilchrist et al., 2013 [15]

Healthy *n* = 30 47 years 112/68 mmHg	Acute 0–2 h	190 mg (200 g spinach)	Rice milk	FMD	2% ↑ after 2 h	Bondonno et al., 2012 [16]

Healthy *n* = 9 25 years 121/71 mmHg	Acute 0–3 h	375 mg (250 mL beetroot juice)	Mineral water	SBP DBP	↓ SBP after 3 h No effect on DBP	Kapil et al., 2010 [17]

Healthy *n* = 14 26 years 106/70 mmHg	Acute 0–6 h–24 h	1437 mg (500 mL beetroot juice)	Mineral water	SBP DBP MAP	↓ SBP ↓ DBP ↓ MAP	Webb et al., 2008 [18]

Healthy *n* = 10 27 years	Acute 0–2 h	1437 mg (500 mL beetroot juice)	Mineral water	FMD	Protection against reperfusion ischemia after 2 h	Webb et al., 2008 [18]

T2DM, type 2 diabetes mellitus; PWV, pulse wave velocity; FMD, flow mediated dilation; SBP, systolic blood pressure; DBP, diastolic blood pressure; MAP, mean arterial pressure.
